# The Mediating Role of Physical Activity and Self-Rated Health in the Association Between Depression and Quality of Life in Older Europeans: An Analysis Differentiated by Sex

**DOI:** 10.3390/jcm13226760

**Published:** 2024-11-10

**Authors:** Marcelo de Maio Nascimento, Adilson Marques, Gerson Ferrari, Élvio Rúbio Gouveia, Andreas Ihle

**Affiliations:** 1 Department of Physical Education, Federal University of Vale do São Francisco, Petrolina 56304-917, Brazil; 2 Swiss Center of Expertise in Life Course Research LIVES, 1227 Carouge, Switzerland; erubiog@staff.uma.pt (É.R.G.); andreas.ihle@unige.ch (A.I.); 3 CIPER, Faculty of Human Kinetics, University of Lisbon, 1649-004 Lisbon, Portugal; amarques@fmh.ulisboa.pt; 4 ISAMB, University of Lisbon, 1649-004 Lisbon, Portugal; 5 Facultad de Ciencias de la Salud, Universidad Autónoma de Chile, Providencia 7500912, Chile; gerson.demoraes@usach.cl; 6 Escuela de Ciencias de la Actividad Física, el Deporte y la Salud, Universidad de Santiago de Chile (USACH), Santiago 9170022, Chile; 7 Department of Physical Education and Sport, University of Madeira, 9000-072 Funchal, Portugal; 8 Laboratory of Robotics and Engineering Systems (LARSYS), Interactive Technologies Institute, 9020-105 Funchal, Portugal; 9 Center for the Interdisciplinary Study of Gerontology and Vulnerability, University of Geneva, 1227 Carouge, Switzerland; 10 Department of Psychology, University of Geneva, 1227 Carouge, Switzerland

**Keywords:** depression, quality of life, self-rated health, physical activity, vulnerability, aging

## Abstract

**Objectives**: This study investigates the mediating role of moderate physical activity (MPA), vigorous physical activity (VPA), and self-rated health (SRH) in the association between depression and quality of life (QoL) in a large sample of Europeans aged 50 and over, differentiated by sex. **Methods**: Data from the 2017 Survey of Health, Ageing and Retirement in Europe were analyzed, including 11,986 individuals (6843 women) aged 50 and older. All information was collected through face-to-face interviews: sociodemographic data, SRH, physical activity levels, depression (EURO-D scale), and QoL (CASP-12). **Results**: Comparatively, women reported a higher prevalence of depression, a lower perception of QoL, and slightly lower levels of SRH, MPA, and VPA. Parallel mediation models revealed, for both sexes, that an increase in VPA levels was more effective in benefiting SRH; and MPA proved to be a better promoter of QoL. When comparing sexes, only the path depression → VPA → QoL showed a significant difference (*p* < 0.001). **Conclusions**: These results provide valuable insights for developing physical activity interventions capable of improving mental health and promoting QoL in older European adults.

## 1. Introduction

Worldwide, approximately 280 million people are suffering from depression [1]. This vulnerability affects mood, causes sadness, fatigue, crying, and loss of appetite, limits the ability to concentrate, alters pleasure or interest in long-term activities, and leads the individual to situations of irritability, including increased suicidal thoughts [2]. Thus, severe symptoms of depression have been considered a global public health problem [3]. It is known that during aging there is an increased risk of developing depression symptoms [4]. Natural aging is a process that increases the likelihood of functional losses and mental disorders [5,6]. These changes negatively impact well-being and the perception of quality of life (QoL) [7]. QoL is a multidimensional construct [8], resulting from the combination of current and past experiences, including opinions and life expectations [9]. QoL represents the judgment of subjective and objective values that an individual has constructed about his or her life [10], a situational view of well-being related to physical, material, social, and emotional aspects [11].

In addition to QoL, self-rated health (SRH) is an important health indicator as it offers an instantaneous view of an individual’s well-being [12]. SRH provides information on health’s biological, mental, functional, and spiritual dimensions. Through SRH, it is possible to understand different perceptions of a person, such as satisfaction, mood, painful symptoms, tiredness, and sadness [13]. SRH can provide insights into health status by reflecting compensatory affective mechanisms resulting from self-esteem [14]. In a practical sense, SRH functions as the reflected discrepancy between low objective health scores and high subjective health scores. In the case of the older population, SRH is useful to detect discomfort problems triggered by sensations associated with age-related chronic diseases [15].

A possible strategy to reduce or avoid symptoms of depression, in addition to promoting the perception of QoL and SRH, is to increase levels of physical activity (PA) [16,17]. PA is considered one of the keys to reducing vulnerability achieving healthy aging [18]. During aging, moderate-to-vigorous physical activity (MVPA) levels are essential to achieve and maintain physical and functional performance, preserve cognitive performance, and promote mental health [19]. The mechanisms underlying caloric expenditure and physical effort favor the functioning of endocrine, immunological, gastrointestinal, muscular, and cognitive functions [20], helping to reduce symptoms of stress, anxiety, and depression [21]. By increasing PA levels, mainly through regular physical exercise, it is possible to reduce the set of pathophysiological mechanisms in the brain that are responsible for depression, such as cytokines, neurotransmitter dysfunction (i.e., serotonin, dopamine, glutamate, gamma-aminobutyric acid), disturbances in neuronal connectivity, brain atrophy, and induction of oxidative stress [22,23]. Consequently, adequate levels of PA reflect positively on well-being, promoting SRH, and the perception of QoL [24].

Over the years, studies have investigated the effects of depression on QoL and SRH in the older population [25,26], as well as the benefits of increased PA levels in reducing depression [21,27] and promoting SRH and QoL [28,29]. Notably, the mechanisms linking depression, QoL, SRH, and PA have not yet been investigated in a comprehensive model that accounts for differences between both sexes. It is known that women are twice as likely to report symptoms of depression compared to men [30]. Throughout life, starting in adolescence, women tend to experience a greater number of stressors than men, such as adverse life events, social and support roles, and genetic and hormonal factors. However, findings regarding women’s greater susceptibility to depression remain inconsistent. [31]. Thus, studies focused on the impact of depression with an emphasis on differentiation by sex may provide a valuable lens through which to examine and expand understanding of the human processes responsible for severe psychopathology. Furthermore, current population-based research has found that men generally report a better QoL than women [32]. Therefore, there are gaps regarding the weight that MPA or VPA exert on SRH, especially when analyzed by sex in the older population. Thus, to address these important gaps, this study aimed to investigate the mediating role of MPA, VPA, and SRH in the association between depression and QoL in a large sample of Europeans aged 50 and over, differentiated by sex.

## 2. Materials and Methods

### 2.1. Study Data

Data comes from the Survey of Health, Ageing and Retirement in Europe (SHARE), specifically from wave 7, conducted in 2017. SHARE is a panel study (biennial cross-national survey on aging), which was carried out for the first time in 2004 [33]. The information was collected through interviews using Computer-Assisted Personal Interviewing (CAPI) in the participants’ homes. The procedures followed the guidelines and ethical standards of the Declaration of Helsinki. Therefore, before participating in the study, all subjects provided informed consent. The SHARE protocol has been reviewed and approved by the Ethics Committee of the University of Mannheim and the Ethics Committee of the Max Planck Society for the Advancement of Science. Detailed information is available on the project’s official website (http://share-eric.eu/, accessed on 22 September 2024), including the methodological details of wave 7.

### 2.2. Participants

**Figure 1:**
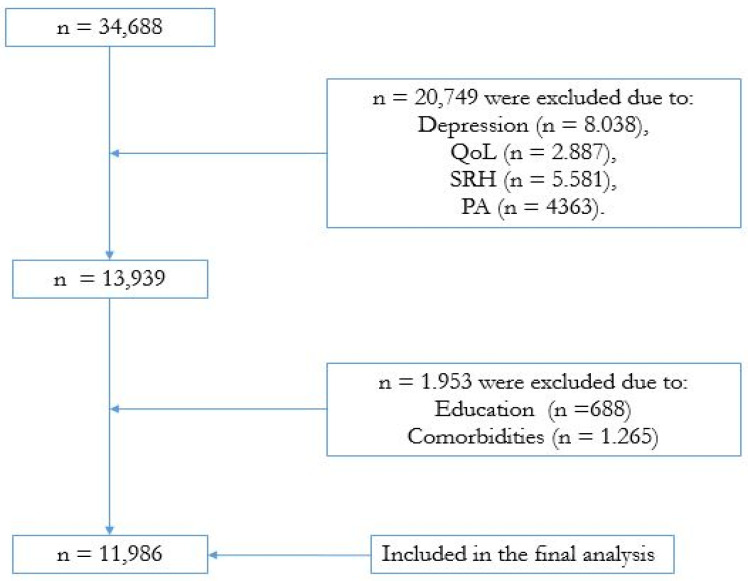
Flowchart of the study design.

In the present study, citizens aged at least 50 were eligible, provided they had complete data from wave 7 (2017). The SHARE database presented 34,688 individuals aged 50 or older (see Figure 1 for a broad overview). However, we excluded participants with missing data for any variable of interest in the study (i.e., depression, QoL, SRH, PA), which determined the inclusion of 11,986 participants (6843 women) with complete data from 12 countries: Austria, Belgium, Czech Republic, Denmark, France, Germany, Greece, Italy, Poland, Spain, Sweden, and Switzerland.

### 2.3. Dependent Variable

#### Quality of Life

The perception of QoL was assessed using the CASP-12 instrument [34], a shorter version of the CASP-19 [35]. The scale includes 12 Likert-type items reflecting four QoL dimensions: control, autonomy, self-actualization, and pleasure. For each of the dimensions, three questions are asked, and each question is evaluated by an increasing scale from 1 to 4. The score for QoL is classified from 12 (lowest) to 48 (highest). The higher the value, the better the perception of the individual’s QoL. The 12-item shortened version of the CASP has stronger measurement properties (Cronbach = 0.87) than the original CASP-19 measure and is recommended for applications.

### 2.4. Independent Variables

#### Depression

We used data from the 12-item EURO-D scale. This instrument is a screening measure applied to identify symptomatology of depression. A detailed description of the 12-item EURO-D, including the validation process, has been reported previously [36]. The cutoff point for classifying the presence of depression symptoms was ≥4 [36]. The scale was considered internally consistent (Cronbach = 0.72), with center-specific values ranging from 0.65 in Dublin to 0.83 in Finland.

### 2.5. Physical Activity

The PA level was obtained by asking: “We would like to know, how often do you do physical activities that require a lot of physical effort, such as sports, heavy housework or a job that requires physical labor?”. Subsequently, this question was associated with two different situations: (1) MPA = activities that required a moderate level of energy (e.g., brisk walking, gardening, or housework), and (2) VPA = vigorous sports or activities (e.g., hiking, sports, carrying heavy loads). The response options offered by the questionnaire were as follows: (1) more than once a week, (2) once a week, (3) up to 3 times a month, and (4) almost never or never. In the present analysis, the last two response options were grouped into “less than once a week”; this procedure was previously published [37].

### 2.6. Self-Rated Health

SRH was obtained through the following question: ‘How is your health in general?’. Eligible responses were ‘very good’, ‘good’, ‘fair’, ‘bad’, and ‘very bad’. For the analysis, we grouped the first two response options (very good and good) in the “good” variable, as well as the last two options (bad and very bad) in the “bad” variable. Thus, three variable values were used to determine the participants’ SRH: good, fair, and bad.

### 2.7. Covariates

Health-related variables that could generate barriers to PA promotion and negatively affect SRH and QoL were considered confounding factors. Thus, according to the variables present in the survey database, we chose to measure body mass index (BMI), level of education, and important chronic-degenerative diseases. All this information was collected by self-report. BMI categories were classified and represented by underweight (BMI < 18 kg/m^2^), normal weight (18 kg/m^2^ ≤ BMI < 25 kg/m^2^); overweight (25 kg/m^2^ ≥ BMI < 30 kg/m^2^), obesity (BMI ≥ 30 kg/m^2^) [38]. The level of education was established based on the International Standard Classification of Education (ISCED) [39]. The different levels were aggregated into three categories: (1) ISCED 0–1, no education or a low level of education; (2) ISCED 2–4, intermediate level of education; and (3) ISCED 5–6, a higher level of education. Participants were also asked to indicate whether they had one or more chronic diseases with a medical report in the past 12 months. A self-report of chronic diseases with medical confirmation is accepted as reliable information on the individual’s general health status [40]. We selected the following diseases: high blood pressure, diabetes, cholesterol, rheumatism, and cancer, as well as neurological diseases that can affect the individual’s autonomy, such as Parkinson’s and Alzheimer’s. This set of chronic diseases is associated with various health problems [41]. Social support was included because it is an important factor in the daily lives of older adults, influencing their mental health [42]. Thus, information about whether or not they have a partner at home was obtained through the binary response (yes/no): “Do you have a partner at home?”.

### 2.8. Statistical Analyses

Data normality was checked using the Kolmogorov–Smirnov test. Descriptive statistics were presented using mean and standard deviation (continuous variables), frequency and percentage (categorical variables). Stratification of the sample was carried out by sex. In the second step, differences between the groups (men, women) were determined using the chi-square test (categorical variables) or Student’s *t*-test (metric variables). Third, we used a multivariate analysis [43] to verify the association between depression, MPA, VPA, SRH (independent variables), and QoL (dependent variable). All independent variables were entered simultaneously into the calculation. The cross-sectional analysis comprised two different models: Model 1 was unadjusted, while Model 2 was adjusted for confounding factors (i.e., age, BMI, education, chronic diseases, and social support). Finally, we performed two parallel and serial mediation analyses, one for the men’s group and the other for women, using Hayes’ PROCESS macro for SPSS, Model 80. This macroprocess uses a bootstrapping method [44], which simultaneously allows the execution of multiple mediations. We consider MPA (*M*_1_) and VPA (*M*_2_) as mediators, sequentially succeeded by the third mediator, which was SRH (*M*_3_). If the association size between the independent variable depression (DEP) and the dependent variable (QoL) did not remain significant, we would consider complete mediation. In this case, the confidence interval should include a zero value [45]. A partial mediation occurred when, after the simultaneous inclusion of the three mediators, the relationship between the independent variable (DEP) and the dependent variable (QoL) became weaker (although remaining significant).

**Figure 2:**
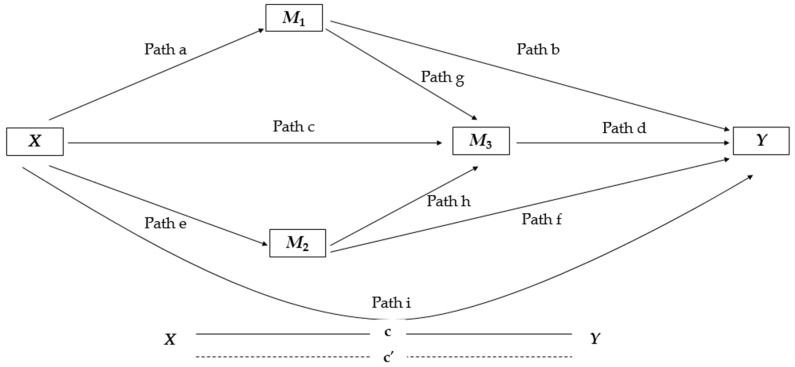
Illustration of the serial mediation parallel model used to explore the role of MPA, VPA, and SRH in mediating the association between depression (DEP) and QoL. Path (a) = association between independent variable DEP (*X*) and mediator MPA (*M*_1_); Path (b) = association between mediator MPA (*M*_1_) and dependent variable QoL (*Y*),;Path (e) = association between independent variable DEP (*X*) and VPA mediator (*M*_2_); Path (f) = association between VPA mediator (*M*_2_) and dependent variable QoL (*Y*); Path (g) = association between MPA mediator (*M*_1_) and SRH mediator (*M*_3_); Path (h) = association between VPA mediator (*M*_2_) and SRH mediator (*M*_3_); Path (c) = association between independent variable DEP (*X*) and SRH mediator (*M*_3_); Path (d) = association between SRH mediator (*M*_3_) and dependent variable QoL (*Y*); and Path (i) represents c’ = direct effect DEP and QoL (*X* − *Y*).

Our trajectory models showed nine direct effects. The complete model (see Figure 2 for better visualization) was composed of four submodels: Models 1 and Model 2 calculated the regressions of each of the parallel mediators *M*_1_ and *M*_2_ in *X*, respectively; Model 3 estimated the regression of *M*_3_ simultaneously on *X*, on *M*_1_ and *M*_2_; and Model 4 determined the regression from *X* to *Y* in *M*_1_, in *M*_2_ and *M*_3_. Specific indirect effects (SIEs) were also estimated. This procedure calculated the product of path coefficients based on a sequence: SIE1 = a×b, SIE2 = a×g×d, SIE3 = c×d, SIE4 = e×f, SIE5 = e×h×d. Moreover, we calculated the total indirect effect = SIE1 + SIE2 + SIE3 + SIE4 + SIE5.

The results of the mediation analyses were presented by standardized regression coefficients (*β*). Results were also presented by proportion mediated (PM), specifically when an indirect mediator indicated a significant effect (95% CI did not contain zero). The PM of the total effect was determined by dividing the indirect effect by the total effect. This technique makes it possible to verify in percentage terms the change in the regression coefficients when a mediating variable is included in the model [46]. In the present study, we calculated and compared two models, one for men and one for women. To assess whether the paths differed significantly between sexes, we compared the respective CIs of both models. Thus, when the CIs did not overlap [47], we assumed a significant difference between men versus women. All procedures were performed using IBM SPSS Statistics version 22 (SPSS Inc., an IBM company, Chicago, IL, USA). The significance level adopted was ᾳ < 0.05.

## 3. Results

### 3.1. Participants’ Characteristics

Regarding the main characteristics of the participants (Table 1), 57.1% were women, and the mean age for the total sample was 71.7 ± 8.13 years. Men showed higher values for BMI (*p* < 0.001) and education (*p* < 0.001). Women indicated a significantly higher prevalence of diabetes (*p* < 0.001), rheumatism (*p* < 0.001), and cancer (*p* = 0.020). Regarding social support, it was 19.8% higher among men (*p* < 0.001). Comparatively, women reported a greater depression score (*p* < 0.001), while men indicated greater scores for QoL, SRH, MPA, and VPA (*p* < 0.001).

### 3.2. Associations Between Depression, MPA, VPA, and SRH with QoL

Table 2 presents the results of the multivariate logistic regression. The model was statistically significant [F(4,5.135) = 668.91; *p* < 0.001; R^2^ = 0.343]. Among men, except for the depression variable, which showed a negative and significant association with QoL (OR = −0.300, *t* = −23.575, *p* < 0.001), all other variables showed a positive and significant association with QoL: MPA (OR = 0.136, *t* = 10.783, *p* < 0.001), VPA (OR = 0.100, *t* = 7.917, *p* < 0.001), and SRH (OR = 0.268, *t* = 20.184, *p* < 0.001). In proportional terms, having high levels of depression represented an odds ratio for reducing QoL by up to 70%. An increase in MPA, VPA, and SRH levels revealed an increased chance of improving QoL by approximately 86.4%, 90%, and 73.2%, respectively (Model 2). Among women, depression showed a negative and significant association with QoL (OR = 0.365, *t* = −33.332, *p* < 0.001), but a positive and significant association with other variables: MPA (OR = 0.145, *t* = 13.688, *p* < 0.001), VPA (OR = 0.055, *t* = 5.270, *p* < 0.001), and SRH (OR = 0.253, *t* = 22.576, *p* < 0.001). Proportionally, having a high level of depression was associated with a 68.9% reduced likelihood of having good QoL. On the other hand, an increase in MPA, VPA, and SRH levels was associated with a likelihood of improving QoL by up to 86.8%, 91.1% and 73.7%, respectively.

After adjusting for confounding factors (i.e., age, BMI, education, chronic diseases, and social support), the model remained significant [F(7,11.674) = 20.171; *p* < 0.001, R^2^ = 0.370]. Thus, men showed a negative and significant association between depression and QoL (OR = −0.311, *t* = −23.344, *p* < 0.001). QoL indicated a positive and significant association with the other independent variables: MPA (OR = 0.132, *t* = 10.459, *p* < 0.001), VPA (OR = 0.089, *t* = 6.965, *p* < 0.001), and SRH (OR = 0.263, *t* = 19,235, *p* < 0.001). Based on the results, we found that an increase in depression represented was likely to reduce QoL by up to 68.9%. At the same time, higher levels of MPA, VPA, and SRH increased the odds of benefiting the QoL by around 86.8%, 91.1%, and 73.7%, respectively. Among women, after controlling for confounding factors (i.e., age, BMI, education, chronic diseases, and social support), there was a negative and significant association between depression and QoL (OR = −0.358, *t* = −32.786, *p* < 0.001). On the other hand, all other independent variables indicated positive and significant associations with QoL: MPA (OR = 0.140, *t* = 12.836, *p* < 0.001), VPA (OR = 0.049, *t* = 4.502, *p* < 0.001), and SRH (OR = 0.227, *t* = 19.059, *p* < 0.001). Thus, proportionally, an increase in depression increased the likelihood of reduced QoL by up to 64.2%. In contrast, an increase in MPA, VPA, and SRH levels increased the odds of QoL by approximately 86.0%, 95.1%, and 77.3%, respectively.

### 3.3. Serial and Parallel Mediation Analysis for Men (n = 5.140)

**Figure 3:**
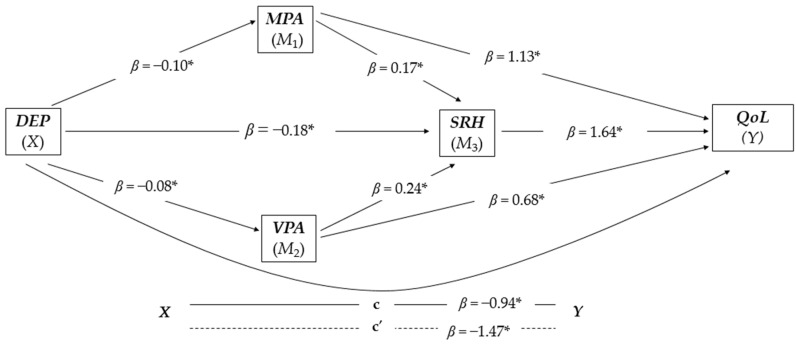
Parallel-serial mediation model of moderate physical activity, vigorous physical activity, and self-rated health in mediating the relationship between depression and quality of life in the group of men (n = 5.140). The products of simultaneous regressions are illustrated by beta (*β*) values. DEP, depression (independent variable); MPA, moderate physical activity; VPA, vigorous physical activity; SRH, self-rated health; QoL, quality of life (dependent variable); * *p* < 0.001.

The overall model was significant and positive [F(1,5138) = 372.2838, *p* < 0.001, R^2^ = 0.367). The three variables, MPA, VPA, and SRH proved to mediate the relationship between depression and QoL (see Figure 3 for a better visualization). In Model 1 that controlled for confounding factors (i.e., age, BMI, education, chronic diseases, and social support), depression was negatively and significantly associated with MPA (*β* = −0.10, *t*(5138) = −19.295, *p* < 0.001). Model 2 was also controlled for confounding factors (i.e., age, education, chronic diseases, and social support), and showed that depression was associated negatively and significantly with VPA (*β* = −0.08, *t*(5138) = −13.602, *p* < 0.001). Model 3 was controlled for confounding factors (i.e., age, BMI, education, chronic diseases, and social support) responsible for the regression of depression on SRH simultaneous with the inclusion of MPA and VPA, and indicated a negative and significant association of depression with SRH (*β* = −0.18, *t*(5138) = −29.1989, *p* < 0.001), and there was a positive and significant association between MPA and SRH (*β* = 0.17, *t*(5138) = 10.426, *p* < 0.001) and also between VPA and SRH (*β* = 0.24, *t*(5138) = 10.1134, *p* < 0.001). Model 4 was also controlled for confounding factors (i.e., age, BMI, education, chronic diseases, and social support), revealing that the three mediators had a positive and significant association with QoL: MPA (*β* = 1.13, *t*(5138) = 10.7828, *p* < 0.001), SRH (*β* = 1.64, *t*(5138) = 20.1842, *p* < 0.001), and VPA (*β* = 0.68, *t*(5138) = 7.9167, *p* < 0.001).

Finally, the direct effect of the model (*x* − *y*) was negative and significant (*β* = −0.94, *t*(5138) = −23.5753, *p* < 0.001). Furthermore, the analysis was based on 10,000 bootstrap samples and revealed negative and significant results for all indirect effect pathways (see Table 3). The proportion of the total effect of depression on QoL mediated by the three mediators was approximately 36.28%. The PM by MPA, SRH, and VPA explained the variance of the association between depression and QoL by up to 7.50%, 20.58%, and 3.96%, respectively.

### 3.4. Serial and Parallel Mediation Analysis for Women (n = 6.843)

**Figure 4:**
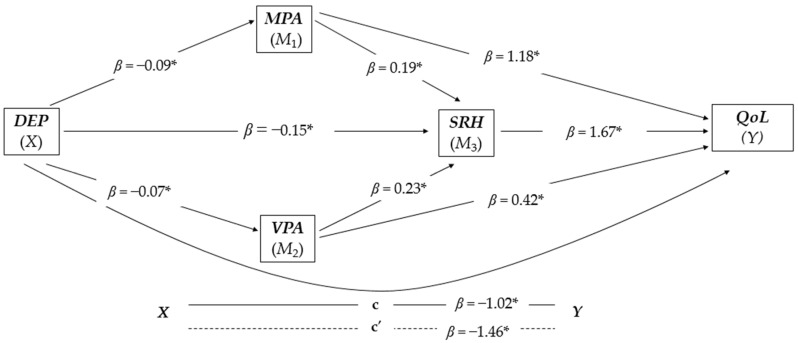
Parallel-serial mediation model of moderate physical activity, vigorous physical activity, and self-rated health in mediating the relationship between depression and quality of life in the group of women (n = 6.842). The products of simultaneous regressions are illustrated by beta (*β*) values. DEP, depression (independent variable); MPA, moderate physical activity; VPA, vigorous physical activity; SRH, self-rated health; QoL, quality of life (dependent variable); * *p* < 0.001.

The overall model was significant and positive [F(1,6840) = 544,2345, *p* < 0.001, R^2^ = 0.312). The three variables, MPA, VPA, and SRH proved to mediate the relationship between depression and QoL (see Figure 4 for a better visualization). Model 1, controlled for confounding factors (i.e., age, BMI, education, chronic diseases, and social support), indicated that depression was negatively and significantly associated with MPA (*β* = −0.09, *t*(6840) = −23.3288, *p* < 0.001). Model 2 was also controlled for confounding factors (i.e., age, BMI, education, chronic diseases, and social support), and indicated that depression was also associated negatively and significantly with VPA (*β* = −0.07, *t*(6840) = −16.0892, *p* < 0.001). In Model 3, controlled for confounding factors (i.e., age, BMI, education, chronic diseases, and social support), SRH was negatively and significantly associated with depression (*β* = −0.15, *t*(6840) = −33.9718, *p* < 0.001), positively and significantly associated with MPA (*β* = 0.19, *t*(6840) = 13.9234, *p* = 0.002), and positively and significantly associated with VPA (*β* = 0.23, *t*(6840) = 18.0335, *p* = 0.001). Model 4 controlled for confounding factors (i.e., age, BMI, education, chronic diseases, and social support), and it showed a positive and significant association between MPA and QoL (*β* = 1.18, *t*(6840) = 13.6878, *p* < 0.001), SRH and QoL (*β* = 1.67, *t*(6840) = 22.5762, *p* < 0.001), and VPA and QoL (*β* = 0.42, *t*(6840) = 5.2705, *p* < 0.001).

The direct effect (*x* − *y*) was negative and significant (*β* = −1.02, *t*(6840) = −34.4678, *p* < 0.001). The proportion of the total effect that SRH had on depression mediated by the three mediators was up to 64.64%. In proportional terms, the total effect of depression on QoL mediated by the three mediators was approximately 30.53%. Moreover, the analysis based on 10,000 bootstrap samples showed negative and significant results for all indirect pathways (see Table 4). Finally, the PM of the variables MPA, SRH, and VPA explained the variance of the association between depression and QoL was up to 8.72%, 17.29%, and 1.99%, respectively.

### 3.5. Differential Mediation Patterns Between Men Versus Women

As shown in Table 4, the total PM by the models for men and women was 36.28% and 30.53%, respectively. Comparatively, women indicated a PM in MPA pathways (SIE 1, SIE 2) that was slightly higher than in men. In contrast, men showed higher PM values in the paths that examined VPA (SIE 4, SIE 5). Among the three mediators, SRH indicated the highest PM, with a higher index among men. Comparison between sex did not show statistical differences (*p* > 0.050) for four paths (i.e., SIE 1, SIE 2, SIE 3). Comparing the two models (men vs. women), only the SIE 4 path (DEP → VPA → QL) showed non-overlapping CIs values (*p* < 0.001). All other paths showed overlapping between CIs values (*p* > 0.050).

## 4. Discussion

This study aimed to investigate the mediating role of MPA, VPA, and SRH in the association between depression and QoL in a large sample of Europeans aged 50 years and older, differentiated by sex. Comparatively, women indicated a higher prevalence of depression and reported slightly lower QoL and SRH than men. For both sexes, MPA, VPA, and SRH demonstrated a mediating role in the relationship between depression and QoL. Comparatively, women indicated slightly higher mediated proportion values in the MPA pathways. In comparison, men presented higher values in the VPA pathways. Regardless of sex, VPA showed greater potential to benefit SRH, while MPA was superior in promoting QoL. Finally, only the path between depression → VPA → QoL revealed a significant difference between the sexes.

Our results confirmed the negative role that depression plays on QoL during aging [48,49]. In line with previous studies, we found that increasing MPA, VPA, and SRH levels may be an effective mechanism to promote QoL in both sexes [50,51] significantly. A recent longitudinal investigation showed that among older European adults, regardless of age group, women reported lower perceived QoL than men, as well as a higher probability of developing symptoms of depression [52]. Over the years, several possible explanations have been provided for sex differences in depression. There are indicators that may lead women to experience more depressive symptoms than men, such as disparities in social and economic dimensions (i.e., financial losses due to retirement or widowhood), less access to health services, and consequently, lower perception of well-being [53]. All of this increases women’s vulnerability to chronic diseases, a greater number of physical limitations, and the use of medications, reflecting negatively on their perception of SRH and QoL [32]. In parallel, genetic and biological issues also increase the likelihood of triggering depressive symptoms in women [30,54]. Consequently, the ability to deal with stressful situations differs between the sexes [55]. Comparatively, women are more likely to admit and complain about dysphoric feelings [56], while men tend to deny their problems, trying to solve them without help, even compensating through alcohol consumption or suicide [56].

A recent review study highlighted a series of factors responsible for the magnitude of the effect of depression on QoL, according to sex [57]. Women were more prone to depression and lower perception of QoL due to the type and number of comorbidities. Consequently, women were more susceptible to somatic and cognitive-affective symptoms than men, in addition to a greater propensity for atypical subtypes of depression, such as hypersomnia, weight gain, and fatigue [58,59]. The negative association between depression and SRH revealed in this study may be a reflection of multifactorial issues responsible for an increase in mental load, leading participants to perceive low well-being [60], and integrating biological, mental, functional, and spiritual factors. Thus, depressed individuals tend to have a low perception of well-being. Consequently, older adults with depressive symptoms have a greater number of comorbidities, which can lead to a greater daily consumption of different types of medications [15], which may obstruct the perception of pleasure or well-being throughout the day.

Our findings showed an inverse relationship between depression and PA levels for both sexes. This association has been well documented in the older European population [26,61]. In old age, depression favors the establishment of a sedentary lifestyle, increasing the risk of developing diseases [62]. On the other hand, changing behavioral factors, more specifically, increasing the weekly frequency of MVPA, presents itself as a strategy capable of reducing or avoiding depressive symptoms [16,63], increasing QoL levels [28]. Depressive symptoms are linked to inflammatory processes (i.e., pro-inflammatory cytokine interleukin-6, intercellular adhesion molecule-1, and acute phase C-reactive protein) and alterations in hormonal, genetic, and neuroimmune mechanisms [64]. On the other hand, MVPA levels have antidepressant potential by inducing in skeletal muscles and releasing into the bloodstream a series of substances that act on cells located in the brain linked to inflammatory processes [65]. In old age, MVPA levels also help maintain body weight, reduce blood pressure, improve insulin resistance, mitigating risks to physical and mental health [66].

Our findings revealed insights into differential mechanisms between men and women regarding weekly PA frequency concerning SRH. In both sexes, VPA (i.e., a frequency greater than once a week) showed greater potential to benefit SRH, while MPA (i.e., a frequency of once a week) was superior in promoting QoL. The findings suggested that both men and women have the same modus operandi to differentiate SRH (in terms of individual well-being) from QoL (in terms of multidimensional health perception) and which weekly PA frequency was superior in promoting SRH or QoL. In the comparison of the two sexes, only the depression → VPA → QoL pathway revealed a significant difference. One explanation for this finding may be the belief that sufficiently active or highly active individuals may have better SRH [24,67]. It is worth noting that, in old age, moderate to vigorous levels of PA can benefit functional fitness [68], which is crucial for the independent management of ADLs [16]. Furthermore, depending on the type of activity, as well as the volume and duration of exercise, moderate to vigorous levels of PA can generate neuroplasticity [19], essential for preserving an autonomous life during aging, consequently benefiting mental health.

### Strengths, Limitations, and Future Directions

First, we present representative information from a large number of European citizens. Second, the findings revealed, in percentage terms, the role of the three mediators in the relationship between depression and QoL, especially the difference between sex concerning the intensity of the PA level. Third, we adjusted the analyses for potentially confounding factors associated with depression symptoms and other biases. Ultimately, our findings can support future actions in emerging healthy aging policies. On the other hand, our study has limitations. First, the cross-sectional data limit interpretations of a causal association. Second, although we included a large sample, the results cannot be generalized to populations outside the European continent. Third, information about PA levels was self-reported and not objectively measured, which may have caused recall bias. Unlike objective measures (accelerometry-based), self-reported measures, such as questionnaires, may lead to overestimating PA levels or intensity. However, this should be comparable for all participants and thus not matter in our correlational study [69]. Fourth, age-related recall problems may have led to misinformation and bias in the analyses.

We suggest that future studies be carried out, and that they stratify the sample by regions of the European continent to compare results based on social, cultural, economic, and political factors. Secondly, an interesting topic of further studies would be investigating the mediating role of PA and SRH in the relationship between QoL and depression symptoms, deepening the analysis according to the remission of depression (spontaneous or due to treatment). Third, when it comes to the intensity of PA and differences between the sexes, there are a series of factors that can influence the MVPA of the older adult population, such as leisure habits, domestic activities, previous exercise experience (e.g., young life and adult), barriers to PA (e.g., motivations, health status, environmental infrastructure). Thus, all of these questions might be included in future research. It is also advisable that future research includes objective measures to assess PA levels, and other mathematical models could be explored, such as complex networks.

## 5. Conclusions

The findings showed that, compared to men, women reported a higher prevalence of depression and lower QoL, SRH, MPA, and VPA. This information can serve as a warning about the mental health of older populations in the countries studied. In a practical sense, regardless of sex, MPA was suggested as the best strategy to promote QoL, while VPA was presented as the best mechanism to benefit SRH. Understanding individual differences between sexes is essential to develop policies capable of monitoring and helping to combat depression during aging. Thus, the information revealed provides valuable subsidies that can help design physical activity interventions specific to each sex, capable of benefiting mental health and promoting the quality of life of the population evaluated.

## Figures and Tables

**Table 1:** Descriptive analysis of the variables studied.

Variable	Total (n = 11,986)	Men (n = 5.143)	Women (n = 6.843)	*p*-Value
**Age (years) (mean ± SD)**	71.7 ± 8.13	72.2 ± 7.65	71.3 ± 8.45	<0.001 *
**BMI**				<0.001 *
Total (mean ± SD)	26.8 ± 4.41	27.1 ± 3.94	26.6 ± 4.73	
Underweight n (f)	120 (1.0)	18 (0.4)	102 (1.5)	
Normal n (f)	4.069 (33.9)	1.499 (29.1)	2.570 (37.5)	
Overweight n (f)	5.046 (42.1)	2.527 (49.1)	2.519 (36.8)	
Obese n (f)	2.454 (20.4)	1.029 (20.0)	1.425 (20.8)	
**Education n (f)**				<0.001 *
ISCED 0–1	3.152 (26.3)	1.163 (22.6)	1.991 (29.1)	
ISCED 2–4	6.172 (51.5)	2.674 (52.0)	3.497 (51.1)	
ISCED 5–6	2.660 (22.2)	1.306 (25.4)	1.355 (19.8)	
**Chronic disease n (f)**				
High blood pressure	5.628 (47.0)	2.416 (47.0)	3.212 (46.9)	0.487
Diabetes	1.786 (14.9)	861 (16.7)	925 (13.5)	<0.001 ^†^
Cholesterol	3.578 (29.9)	1.506 (29.3)	2.072 (30.3)	0.124
Rheumatism	1.229 (10.3)	345 (6.7)	884 (12.9)	<0.001 ^†^
Cancer	517 (4.3)	245 (4.8)	272 (4.0)	0.020 ^†^
Parkinson	130 (1.1)	76 (1.5)	54 (0.8)	<0.001 ^†^
Alzheimer	197 (1.6)	81 (1.6)	116 (1.7)	0.331
**Partner in the house, n (f)**				<0.001 ^†^
Yes, n (%)	8.306 (69.3)	4.145 (80.6)	4.161 (60.8)	
No, n (%)	3.680 (30.7)	998 (19.4)	2.682 (39.2)	
**DEP**				<0.001 *
Total (mean ± SD)	2.3 ± 2.26	1.9 ± 1.98	2.7 ± 2.40	
Not depressed n (f)	8.851 (73.8)	4.193 (81.5)	4.658 (68.1)	
Little depressive n (f)	2.387 (19.9)	777 (15.1)	1.610 (23.5)	
Fairly depressed n (f)	659 (5.5)	154 (3.0)	505 (7.4)	
Very depressing n (f)	89 (0.7)	19 (0.4)	70 (1.0)	
**QoL**				
Total (mean ± SD)	37.0 ± 6.45	37.6 ± 6.21	36.6 ± 6.59	<0.001 *
**SRH**				
Total (mean ± SD)	2.8 ± 1.00	2.9 ± 1.01	2.8 ± 0.99	0.007 *
Bad n (f)	1.035 (8.6)	436 (8.5)	599 (8.8)	
Fair n (f)	3.337 (27.8)	1.383 (26.9)	1.954 (28.6)	
Good n (f)	7.613 (63.6)	3.323 (64.6)	4.290 (62.7)	
**MPA n (f)**				<0.001 *
<Once week	2.177 (18.2)	801 (15.6)	1.376 (20.1)	
Once week	1.723 (14.4)	740 (14.4)	983 (14.4)	
>Once week	8.086 (28.7)	3.602 (70.0)	4.484 (65.5)	
**VPA n (f)**				<0.001 *
<Once week	6.756 (18.2)	2.709 (52.7)	4.047 (59.1)	
Once week	1.784 (14.9)	742 (14.4)	1.042 (15.2)	
>Once week	3.446 (28.7)	1.690 (32.9)	1.754 (25.6)	

BMI, body mass index; DEP, depression; f, frequency (%); QoL, quality of life; SRH, self-rated health; MPA, moderate physical activity; VPA, vigorous physical activity; SD, standard deviation; ^†^ Chi-square test *p* < 0.001; * Mann–Whitney U test *p* < 0.001.

**Table 2:** Multivariate analysis for the association between depression, moderate physical activity, vigorous physical activity, and self-rated health with quality of life.

Variable		Model 1			Model 2	
	OR	95% CI	*p*-Value	OR	95% CI	*p*-Value
**Men**						
DEP (n)	0.300	(−1.018, −0.862)	<0.001	0.311	(−1.023, −0.865)	<0.001
MPA (n)	0.136	(0.922, 1.332)	<0.001	0.132	(0.897, 1.311)	<0.001
VPA (n)	0.100	(0.516, 0.856)	<0.001	0.089	(0.439, 0.783)	<0.001
SRH (n)	0.268	(1.0480, 1.798)	<0.001	0.263	(1.0446, 1.774)	<0.001
**Women**						
DEP (n)	0.365	(−1.068, −0.949)	<0.001	0.358	(−1.050, −0.931)	<0.001
MPA (n)	0.145	(1.017, 1.357)	<0.001	0.140	(0.972, 1.323)	<0.001
VPA (n)	0.055	(0.267, 0.584)	<0.001	0.049	(0.209, 0.532)	<0.001
SRH (n)	0.253	(1.523, 1.813)	<0.001	0.227	(1.344, 1.652)	<0.001

DEP, depression; MPA, moderate physical activity; VPA, vigorous physical activity; SRH, self-rated health. Model 1—unadjusted. Model 2—adjusted for age, BMI, education, chronic diseases, and social support.

**Table 3:** Relationship between depression, moderate physical activity, vigorous physical activity, and self-rated health with quality of life in men (n = 5.140).

SIE	Pathways Key	Indirect Effect (95% CI)	PM
	Total (Model)	−0.5354 (−0.5804, −0.4897)	36.28%
SIE 1 = a×b	DEP → MPA → QoL	−0.1107 (−0.1356, −0.0868)	7.50%
SIE 2 = a×g×d	DEP → MPA → SRH → QoL	−0.0282 (−0.0358, −0.0215)	1.91%
SIE 3 = c×d	DEP → SRH → QoL	−0.3037 (−0.3388, −0.2696)	20.58%
SIE 4 = e×f	DEP → VPA → QoL	−0.0584 (−0.0746, −0.0424)	3.96%
SIE 5 = e×h×d	DEP → VPA → SRH → QoL	−0.0344 (−0.0417, −0.0279)	2.33%

SIE, specific indirect effect; DEP, depression; MPA, moderate physical activity; VPA, vigorous physical activity; SRH, self-rated health; QoL, quality of life; PM, proportion mediated.

**Table 4:** Relationship between depression, moderate physical activity, vigorous physical activity, and self-rated health with quality of life in women (n = 6.843).

SIE	Pathways Key	Indirect Effect (95% CI)	PM
	Total (Model)	−0.4465 (−0.4804, −0.4131)	30.53%
SIE 1 = a×b	DEP → MPA → QoL	−0.1276 (−0.1276, −0.0903)	8.72%
SIE 2 = a×g×d	DEP → MPA → SRH → QoL	−0.0297 (−0.0354, −0.0245)	2.03%
SIE 3 = c×d	DEP → SRH → QoL	−0.2529 (−0.2803, −0.2249)	17.29%
SIE 4 = e×f	DEP → VPA → QoL	−0.0291 (−0.0409, −0.0183)	1.99%
SIE 5 = e×h×d	DEP → VPA → SRH → QoL	−0.0265 (−0.0316, −0.0219)	1.81%

SIE, specific indirect effect; DEP, depression; MPA, moderate physical activity; VPA, vigorous physical activity; SRH, self-rated health; QoL, quality of life; PM, proportion mediated.

## Data Availability

Data may be accessed through becoming a registered user with the Survey of Health, Ageing and Retirement in Europe (via www.share-project.org) (accessed on 21 July 2023).
